# Implementation of broad screening with Ebola rapid diagnostic tests in Forécariah, Guinea

**DOI:** 10.4102/ajlm.v6i1.484

**Published:** 2017-03-31

**Authors:** Frantz Jean Louis, Jennifer Y. Huang, Yacouba K. Nebie, Lamine Koivogui, Gayatri Jayaraman, Nadine Abiola, Amanda Vansteelandt, Mary C. Worrel, Judith Shang, Louise B. Murphy, David L. Fitter, Barbara J. Marston, Lise Martel

**Affiliations:** 1 Centers for Disease Control and Prevention, Port-au-Prince, Haiti; 2 Centers for Diseases Control and Prevention, Atlanta, Georgia, United States; 3 World Health Organization, Conakry, Guinea; 4 Institut National de Sante Publique, Conakry, Guinea; 5 Centers for Diseases Control and Prevention, Kinshasa, Congo; 6 Centers for Diseases Control and Prevention, Yaoundé, Cameroon; 7 Centers for Diseases Control and Prevention, Conakry, Guinea

## Abstract

**Background:**

Laboratory-enhanced surveillance is critical for rapidly detecting the potential re-emergence of Ebola virus disease. Rapid diagnostic tests (RDT) for Ebola antigens could expand diagnostic capacity for Ebola virus disease.

**Objectives:**

The Guinean National Coordination for Ebola Response conducted a pilot implementation to determine the feasibility of broad screening of patients and corpses with the OraQuick^®^ Ebola RDT.

**Methods:**

The implementation team developed protocols and trained healthcare workers to screen patients and corpses in Forécariah prefecture, Guinea, from 15 October to 30 November 2015. Data collected included number of consultations, number of fevers reported or measured, number of tests performed for patients or corpses and results of confirmatory RT-PCR testing. Data on malaria RDT results were collected for comparison. Feedback from Ebola RDT users was collected informally during supervision visits and forums.

**Results:**

There were 3738 consultations at the 15 selected healthcare facilities; 74.6% of consultations were for febrile illness. Among 2787 eligible febrile patients, 2633 were tested for malaria and 1628 OraQuick^®^ Ebola RDTs were performed. A total of 322 OraQuick^®^ Ebola RDTs were conducted on corpses. All Ebola tests on eligible patients were negative.

**Conclusions:**

Access to Ebola testing was expanded by the implementation of RDTs in an emergency situation. Feedback from Ebola RDT users and lessons learned will contribute to improving quality for RDT expansion.

## Introduction

The Ebola virus disease (EVD) outbreak in Guinea started in December 2013 and affected 25 of the 34 administrative prefectures of the country.^1^ Laboratory-enhanced surveillance is critical for rapid detection of the potential re-emergence of EVD. The capacity of the laboratory system in Guinea is characterised by poor infrastructure, insufficient numbers of qualified personnel, lack of instrumentation and a limited quality assurance system. To support the diagnostic capacity to adequately respond to the EVD outbreak, multiple countries and organisations deployed mobile laboratories and/or diagnostic equipment for rapid detection of the Ebola virus. While these laboratories were critical to ensuring prompt and effective case management during the response,^2^ many are downsizing or ending operations. Yet the need for consistent and ongoing capacity for diagnosis of EVD still exists in Guinea. Testing capacity is limited throughout the country, and Guinea currently lacks a timely and reliable specimen referral system for the safe transfer of specimens to centralised testing facilities.

Current EVD diagnosis relies heavily on reverse transcription polymerase chain reaction (RT-PCR) technology. While RT-PCR is highly sensitive and specific and is considered the gold standard for EVD diagnosis, it requires skilled technicians and an appropriate laboratory infrastructure, including stable power supply, controlled temperature, and appropriate biosafety procedures. Rapid diagnostic tests (RDTs), such as lateral flow assays that detect Ebola antigens, could address many of the challenges of relying on laboratory-based RT-PCR. Compared to PCR, antigen-based RDTs are better adapted to use in the field, can be designed to require limited or no cold chain, require less training and equipment and can provide results in minutes.^3^ In November 2014, the World Health Organization (WHO) issued a call for ‘rapid, sensitive, safe and simple Ebola diagnostic tests’ adapted for severely resource-constrained settings.^4^ In response, several companies developed antigen-based RDTs. As of late 2015, two of these had received regulatory approval by the WHO or the US Food and Drug Administration for Emergency Use Authorization. The ReEBOV™ Antigen Rapid Test (Corgenix, Broomfield, Colorado, United States) was approved by WHO and was issued an Emergency Use Authorization for use with whole blood by the US Food and Drug Administration in February 2015. The OraQuick^®^ Ebola Rapid Antigen test (OraQuick^®^ Ebola RDT; OraSure Technologies, Inc., Bethlehem, Pennsylvania, United States) received an Emergency Use Authorization from the US Food and Drug Administration in July 2015 for whole blood testing.^5,6,7^ The OraQuick^®^ Ebola RDT has a manufacturer-reported sensitivity of 84% (95% confidence interval [CI]: 63.92–95.46) and a specificity of 98.0% (95% CI: 89.35–99.95) for whole blood. By late 2015, there were no data on the performance of the OraQuick^®^ Ebola RDT for oral fluid and overall very limited data and experience with field implementation of the rapid tests.

Although the yield of the Ebola RDT was expected to be low (given the low prevalence of Ebola at this stage of the epidemic), the identification of an unknown Ebola transmission chain was considered a high priority, given the duration and mortality rate of the epidemic. The implementation of Ebola RDTs in this emergency situation would allow broader screening of a population that had limited access to Ebola RT-PCR testing and a high prevalence of diseases with Ebola-like symptoms, such as malaria.

The Ebola response coordination in Guinea determined that while rapid tests could play an important role in surveillance for EVD, additional information about their performance was needed. Laboratories in Guinea conducted additional testing of several rapid tests, including the OraQuick^®^ Ebola RDT, to improve local familiarity with the tests and confirm their performance characteristics in terms of sensitivity and specificity (unpublished data). In collaboration with the WHO, the Guinean Red Cross (RC), the US Centers for Disease Control and Prevention, and the Guinean National Institute of Public Health developed plans to evaluate the potential to screen more broadly with the OraQuick^®^ Ebola RDT, based on its performance results and the Emergency Use Authorization from the US Food and Drug Administration. Forécariah prefecture was chosen for the OraQuick^®^ Ebola RDT field evaluation pilot, given the high impact of EVD in this prefecture in 2015 and the prefecture’s contiguity with Sierra Leone. Forécariah is located in western Guinea, with an estimated population of 244 649 people.^8^ The prefecture was one of the most highly affected in Guinea during the 2013–2015 West African EVD epidemic; the cumulative incidence of EVD in Forécariah was nearly six times greater than that of Guinea overall (Forécariah = 198 cases per 100 000 population; Guinea = 36 cases per 100 000 population).^9^ As one of the most affected areas, Forécariah was included in a March 2015 Presidential Health emergency declaration that required secure burial practices for all deaths.^10^ During initial planning for the evaluation, EVD transmission had been controlled in Forécariah, and the prefecture was considered an example of a recently-affected area. However, EVD was reintroduced in Forécariah in September 2015, resulting in limited additional transmission.^9^ During the epidemic, the response infrastructure in Forécariah grew to meet the prefecture’s increasing case load, and by April 2015 included an Ebola Treatment Unit (ETU) and a laboratory with RT-PCR capacity (K-Plan laboratory).^11^ Here, we report the initial results of the pilot implementation of the OraQuick^®^ Ebola RDT in Forécariah Prefecture from 15 October to 30 November 2015. Our objectives were to document the implementation process and to describe the results of Ebola RDTs, initial feedback from RDT users and major barriers to implementation.

## Methods

### Ethical considerations

The protocol was approved as a non-research programme evaluation activity at the US Centres for Disease Control and Prevention. The activity was authorised by the Guinean National Coordination for Ebola Response.

### Planning

**FIGURE 1:**
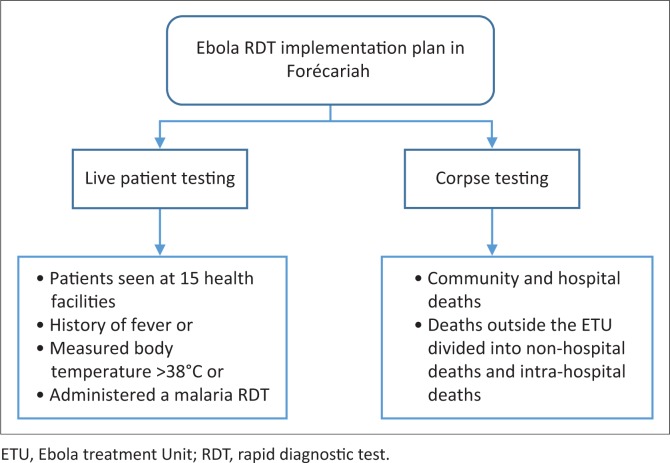
OraQuick^®^ Ebola RDT pilot study implementation strategies in Forécariah, Guinea, 15 October to 30 November 2015.

Two strategies were developed for the Ebola RDT implementation pilot plan: testing on live patients and testing corpses (Figure 1). All partners involved in laboratory-based surveillance activities participated in the elaboration of the OraQuick^®^ Ebola RDT implementation plan. The laboratory cluster working group for the Ebola response, led by the director of the Guinean National Institute of Public Health, coordinated the activities and helped define roles and responsibilities for all stakeholders. The WHO, the Guinean National Institute of Public Health and the US Centers for Disease Control and Prevention developed the training materials; RC volunteers were responsible for screening corpses from both the community and hospitals and for ensuring safe burials, while the K-Plan Laboratory in Forécariah played a key role in PCR testing for confirmation of OraQuick^®^ Ebola RDT results. Prefectural epidemiologists from the US Centers for Disease Control and Prevention, the WHO and the Guinean Ministry of Health were responsible for data collection and for supervising the selected health facilities to monitor the OraQuick^®^ Ebola RDT implementation in the field.

### Health facility selection

**FIGURE 2:**
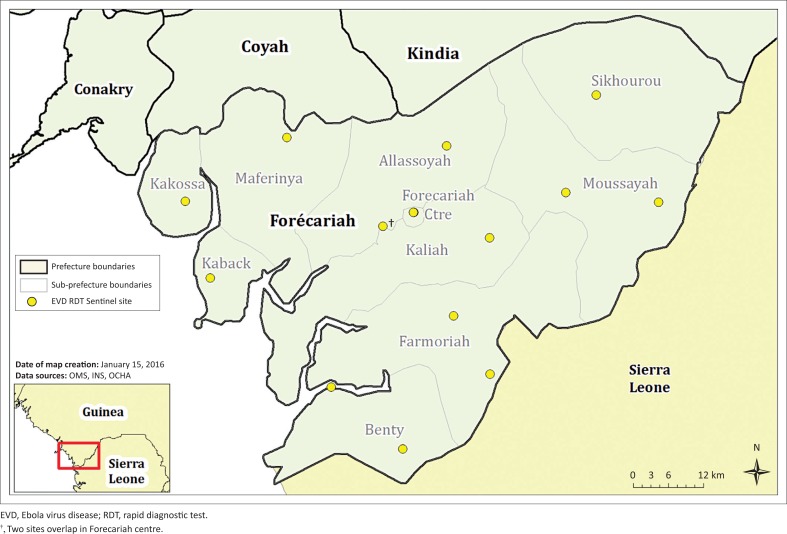
Map of health facilities selected for the OraQuick^®^ Ebola RDT pilot in Forécariah†.

Initially, all health posts (*n* = 36) and health centres (*n* = 10) in Forécariah were designated to implement the RDT pilot. However, to improve the feasibility of the pilot, 15 sites were ultimately selected based on location and patient volume. At least one health facility was selected in each of the nine sub-prefectures of Forécariah. Additional facilities were chosen along the border with Sierra Leone (Figure 2).

### Eligibility criteria and patient and corpse management

The eligibility criteria for testing were designed to be sensitive and to capture all patients with possible unknown EVD contact (Figure 1). Given the low sensitivity of the OraQuick^®^ Ebola RDT at low viral load (initial phase of illness), known and monitored contacts of persons with EVD were not eligible for RDTs. Instead, any monitored contact who developed symptoms compatible with Ebola was to be transferred to an ETU for evaluation. Patients with a temperature > 38 °C or with history of fever in the 48 hours preceding consultation were eligible for the OraQuick^®^ Ebola RDT. Given the similarities between Ebola and malaria symptoms (e.g., fever, chills, body aches, nausea, and vomiting), evaluation procedures specified concurrent testing with a malaria RDT.^12^ Given their identical testing criteria, the malaria RDT is a valuable comparator for the utilisation rate of Ebola RDTs and for identifying any Ebola RDT-specific implementation issues. Patients with a positive Ebola RDT were referred to an ETU for RT-PCR confirmation using the Real Star Filovirus Screen RT-PCR Kit 1.0 (Altona Diagnostics, Hamburg, Germany), and the Light-Cycler 480 (Roche Molecular Diagnostics, Pleasanton, California, United States). All community and hospital deaths were also eligible for screening using OraQuick^®^ Ebola RDT. Because the OraQuick^®^ Ebola RDT had not been approved for swabs, a second swab was also collected for RT-PCR confirmation and programme decisions. For example, the decision whether to trace the contacts of the deceased was driven by the RT-PCR results. These RDT data on swabs were sent to WHO and the US Centers for Disease Control and Prevention for validation. By presidential edict, all burials were secured by the RC without waiting for test results.

### Training materials

A set of presentations was prepared to cover the following topics: protocol for the implementation of the Ebola RDT and eligibility criteria to screen patients and corpses; principles and use of the OraQuick^®^ Ebola RDT; quality assurance for using the OraQuick^®^ Ebola RDT; communication practices around testing results; use of personal protective equipment; waste management; data collection; and supervision tools. The training was designed for healthcare workers (i.e., physicians, nurses, health agents) and for RC volunteers.

### Communication around Ebola rapid diagnostic tests

Healthcare workers were given talking points to help them explain and inform patients about the differences between the RDTs for malaria and Ebola and the Ebola RT-PCR test, as well as the steps that would be taken in response to a test result. Communication around swabs of corpses focused on targeted messaging for the RC volunteers to give to the families of the deceased and for healthcare workers to give to patients. Because secure burials were required regardless of test results, and concurrent PCR testing was done for all deaths, it was agreed among partners not to provide the results of negative RDTs to families.

### Personal protective equipment and waste disposal

Use of personal protective equipment, including safety goggles and/or face shields, masks or respiratory equipment, disposable gowning, boots and gloves, was recommended for performing Ebola RDTs. Used RDTs were decontaminated in 3% chlorine solution before disposal. At the 15 selected health facilities, used personal protective equipment was either incinerated or buried deeply, depending on available resources for waste management. Used personal protective equipment from the burial teams was transported safely to an RC facility for incineration and burial.

### Test kit distribution and quality control

Ebola RDT kits and consumables were distributed to the selected health facilities during the training. Data on stock management and replenishment of kits and consumables were collected at each supervision visit. The protocol called for all positive Ebola RDTs and 10% of negative samples from live patients to be selected by convenience sampling for confirmatory testing by RT-PCR. All samples collected from corpses were tested by RT-PCR.

### Health facilities supervision and data collection

A standardised supervision checklist was developed for use at initial and follow-up visits to evaluate the following elements at each selected facility: laboratory infrastructure; personnel competencies; documentation; and storage and stock management capacity. The checklist was also designed to evaluate specimen and waste management in accordance with recommended biosafety standards, and the basic elements of quality assurance (proper documentation, positive and negative controls, availability of standard operating procedures, and respect of RDT reading time). Following initial training, prefectural epidemiologists conducted weekly supervision and collected data on the OraQuick^®^ Ebola RDT implementation. Data were collected by phone when logistical constraints prevented the epidemiology team from visiting the health facilities. During the epidemiologists’ visits, clinic registries were reviewed for several variables of interest (i.e., number of consultations; number of fevers [reported by patient as ‘I have a fever’ or ‘I feel feverish’ (recorded), number of fevers (measured as > 38 °C)]; number of patients tested with the OraQuick^®^ Ebola RDT; number of corpses screened with the OraQuick^®^ Ebola RDT; number of specimens confirmed by RT-PCR; number of malaria RDTs used; and number of positive malaria RDTs). Teams also collected informal qualitative information from practitioners about their experiences with the Ebola RDT and lessons learned, through forums with healthcare workers and RC volunteers and discussions in the field during this pilot.

## Results

### Pre-implementation

During the initial training in September 2015, 166 participants were trained (101 healthcare workers and 65 RC volunteers) over three days.

### Live patient rapid diagnostic tests

A total of 28 patients were tested for EVD in Forécariah during the six weeks prior to the pilot. Between 15 October 2015 and 30 November 2015, there were 3738 consultations at the 15 selected healthcare facilities. Of these, 74.6% (*n* = 2787) of consultations were for febrile illness (reported or measured), of which 58.4% (*n* = 1628 of 2787) were screened for EVD (Table 1). Only 14% (*n* = 393 of 2787) of the reported fever cases had an actual measured fever of over 38°C. During the same period of time, 94.5% (*n* = 2633 of 2787) of reported fevers were tested for malaria.

**TABLE 1:** Key results of the Ebola rapid diagnostic test pilot study at 15 sites in Forécariah, Guinea, 15 October to 30 November 2015.

Variables	Across sentinel sites		

	Total (%)	Median	Interquartile Range
	**Live patients**		
Number of consultations	3738	176	124–353
Number of fevers (recorded or measured)	2787 (74.6%)	128	94–247
Number of fevers (measured > 38 °C)	393 (10.5%)	15	11.5–28
Number of patients tested for Ebola RDT	1628 (58.4%)	94	56.5–129.5
Number of positive Ebola RDT	0†	-	-
Number of negative Ebola RDT	1627	-	-
Number of negative Ebola RDT confirmed by RT-PCR	163	-	-
Number of patients tested for malaria RDT	2633 (94.5%)	127	97.5–241
Number of positive malaria RDT	1771 (67.3%)	110	68–165.5
Proportion of Ebola RDT to malaria RDT	0.62	0.76	0.53–0.96
	**Corpses**		
Number of corpses screened by Ebola RDT	322	30	18.5–38
Number of corpses screened by Ebola RDT confirmed by RT-PCR	322	30	18.5–38

RDT, rapid diagnostic test; RT-PCR, reverse transcription polymerase chain reaction.

† , One case tested positive by the RDT but was confirmed negative by the reference method, RT-PCR.

During the evaluation, there was one false positive Ebola RDT, which was from a person who was a high-risk contact of a patient confirmed to have EVD who had initially refused transfer to an ETU, but presented to one of the health facilities participating in the pilot. She was tested by RDT independent of the evaluation protocol and then immediately transferred to an ETU, where the result was confirmed negative by RT-PCR. A total of 163 (10%) negative RDT test results were confirmed negative by RT-PCR as part of the quality assurance process put in place. The ratio of Ebola RDTs to malaria RDTs averaged 0.62 (range 0.19 to 1.08). The proportion of positive malaria RDTs averaged 67.3% (*n* = 1771) of all patients seeking care for febrile illness.

### Feedback from healthcare workers

The gap between malaria RDT and Ebola RDT use may be explained by healthcare workers’ misunderstanding of the testing algorithm (some healthcare personnel believed that they were only to conduct the OraQuick^®^ Ebola RDT when the patient temperature was at least 38 °C) and also by temporary stock-outs of OraQuick^®^ Ebola RDT kits. Other reasons why the Ebola RDT was not conducted included the following: patient declined; healthcare personnel forgot to conduct the test or decided that only a test for malaria was indicated; or the person responsible for conducting the test was not at work at that time. Based on reports from workers at multiple sites, healthcare personnel did not regularly inform the patient when administering the Ebola RDT. The most commonly-cited reason for not informing the patient was concern that patients would refuse EVD testing. Some healthcare workers were concerned that if the community became aware that the local clinic was testing for EVD, the patients would avoid the clinic because of negative associations with the disease. Several healthcare workers also reported that patients coming from villages that had been affected by EVD were more receptive to the test than those coming from unaffected villages.

Logistical constraints and the high turnover of implementing partners in the field hindered regular supervision of the healthcare facilities, which in turn affected the completeness of data collection, distribution of Ebola RDT kits and controls, onsite technical assistance and overall quality assurance.

### Community deaths

A total of 332 deaths were reported during the study period and 97% (*n* = 322) were tested for EVD. All 322 corpses screened with OraQuick Ebola RDT were negative. These samples were all confirmed negative by RT-PCR. No healthcare workers or RC volunteers reported problems related to handling or testing using OraQuick^®^ Ebola RDT. During a refresher training session in late November, RC volunteers reported no difficulties with families of the deceased resulting from use of the RDT during secure burials.

## Discussion

Neither healthcare providers nor RC volunteers reported serious problems performing the OraQuick^®^ Ebola RDT. During the pilot period, the broader testing criteria for the Ebola RDT increased the number of people being tested for EVD, even though only a little more than half of the eligible patients were tested. The number of live patients tested for EVD in Forécariah increased more than 20-fold and 97% of reported deaths were screened. Thus, RDTs appear to offer an important tool for expansion of surveillance for EVD, in line with WHO recommendations.^13^ Importantly, there were no serious problems with false positive results; about 10% of negative samples were collected randomly and retested using RT-PCR, with all results confirmed negative. In the one case, when the initial screening test was positive, either falsely or because of incorrect techniques, it was straightforward to conduct follow-up testing with RT-PCR and resolve the situation.

While all febrile patients should have been eligible for both malaria and Ebola RDT tests, most febrile patients had a malaria RDT done, but only about half of febrile patients were tested for EVD. This gap is likely explained by a combination of RDT user error (misunderstanding or forgetting the RDT protocol), logistics (stock out, lack of personnel), or patient refusal. The utilisation rate could be improved with regular supervision, improved job aids, onsite technical assistance and better support for RDT stock management. The usage and positivity rate of the malaria RDT were higher in the 15 health facilities than reported previously in Guinea.^14^ The high proportion of positive malaria tests could reflect season or altered patterns of care-seeking behavior.^14^

The biggest challenges for the implementation of OraQuick^®^ Ebola RDT in Forécariah were data collection, poor RDT stock management causing frequent stock-outs, and logistic and environmental constraints. The travel time from the centre of Forécariah to the health facilities was as much as several hours, depending on the weather and road conditions. The inconsistent connectivity, either through cell phone reception or electricity, was a barrier to receiving data by phone or using electronic transmission. Some of the health facilities were staffed by only one person, and both data collection and management require time and training. Finally, implementing partners in the field had high staff turnover, which hampered regular monitoring and evaluation of the project, as well as site supervision, implementation of the standardised supervision checklist, kits and control distribution, and technical assistance that would improve tracking of implementation progress, as well as measurable quality assurance in the programme.

Broader implementation of the RDT will have to address multiple health communications issues, including the stigma attached to EVD, public mistrust of facilities performing Ebola testing, and pre- and post-test counseling for patients undergoing an Ebola RDT. Lessons learned from HIV RDT implementation will be a useful model. Costs for the OraQuick^®^ Ebola RDT are currently high, which may limit more widespread roll out of the test and further validation of test performance is necessary.

### Limitations

The main limitation of this study is the lack of follow-up on tools put in place to ensure quality; positive and negative controls were merely implemented, and supervision to ensure that standard operating procedures are followed was lacking. Continuous training will also be needed to integrate Ebola RDT as part as a routine testing for eligible patients in Guinea.

### Conclusions

The use of RDTs facilitated a marked increase in the numbers of suspected patients and corpses tested for Ebola in Forécariah, contributing to the critical objective of maintaining vigilant surveillance for Ebola in the context of the recently controlled epidemic. The implementation programme was made possible by the high political commitment of the national coordination for the control of EVD, as well as the support from the Guinean National Institute of Public Health and all stakeholders. The comparison of Ebola RDT and malaria RDT utilisation allowed field teams to identify Ebola RDT specific issues. Feedback from Ebola RDT users contributed to the development of new protocols for improved quality assurance and project tracking during the expansion to other testing sites. Lessons learned from this pilot will guide the expansion of OraQuick^®^ Ebola RDT throughout the country to support surveillance, to identify potential undetected cases and prevent future outbreaks.

BOX 1 Lessons Learned.

- Ebola Rapid Diagnostic Testing can offer an important tool for expansion of surveillance for EVD in settings where access to molecular testing is limited.
- Continuous training and supervision are critical to ensure quality of Ebola Rapid Diagnostic Testing in the field.
- Integration of Ebola Rapid Diagnostic Testing into existing surveillance infrastructure and strategies such as surveillance for malaria will improve data flow and acceptance of the testing.

## References

[1] World Health Organization Emergencies preparedness, response: Ebola maps 2015 [page on the Internet]. c2015 [cited 2015 Dec]. Available from: https://www.who.int/csr/disease/ebola/maps-2015/en/

[2] Diers J, Kouriba B, Ladan Fofana L, et al [Mobile laboratories for rapid deployment and their contribution to the containment of emerging diseases in Sub-Saharan Africa, illustrated by the example of Ebola virus disease] [article in French]. Med Sante Trop. 2015;25(3):229–233. https://doi.org/10.1684/mst.2015.0485 26446739 10.1684/mst.2015.0485

[3] Nouvellet P, Garske T, Mills HL, et al The role of rapid diagnostics in managing Ebola epidemics. Nature. 2015;528(7580):S109–116. https://doi.org/10.1038/nature16041 26633764 10.1038/nature16041 PMC4823022

[4] World Health Organization Urgently needed: rapid, sensitive, safe and simple Ebola diagnostic tests [page on the Internet]. c2014 [cited 2015 Dec]. Available from: https://www.who.int/mediacentre/news/ebola/18-november-2014-diagnostics/en/

[5] Broadhurst MJ, Kelly JD, Miller A, et al ReEBOV Antigen Rapid Test kit for point-of-care and laboratory-based testing for Ebola virus disease: a field validation study. Lancet. 2015;386(9996):867–874. https://doi.org/10.1016/S0140-6736(15)61042-X 26119838 10.1016/S0140-6736(15)61042-X

[6] World Health Organization WHO Emergency Use assessment and listing for Ebola virus disease IVDs: public report [document on the Internet]. c2015 [cited 205 Dec]. Available from: https://www.who.int/diagnostics_laboratory/procurement/150219_reebov_antigen_rapid_test_public_report.pdf

[7] US Food and Drug Administration Emergency Use Authorizations [page on the Internet]. c2015 [cited 2015 Dec]. Available from: https://www.fda.gov/MedicalDevices/Safety/EmergencySituations/ucm161496.htm

[8] Institut National de la Statistique Répartition population et densité par région et prefecture [page on the Internet]. c2015 [cited 2015 Dec 21]. Available from: https://www.stat-guinee.org/index.php/statistiques/donnees-structurelles/demographie/31-population-densite-region-prefecture

[9] World Health Organization Ebola data and statistics [page on the Internet]. c2015 [cited 2015 Dec]. Available from: https://apps.who.int/gho/data/view.ebola-sitrep.ebola-summary-20151211?lang=en

[10] Bureau de Presse de la Présidence de Guinee Ebola: état d’urgence renforcé pour les préfectures de Forécariah, Coyah, Dubréka, Boffa et Kindia pour une période de 45 jours [page on the Internet]. c2015 [cited 2015 Dec]. Available from: https://guineelive.com/2015/03/29/ebola-etat-durgence-renforce-pour-les-prefectures-de-forecariah-coyah-dubreka-boffa-et-kindia-pour-une-periode-de-45-jours/

[11] Croix Rouge Francaise Ebola: la riposte se déplace à Forécariah [page on the Internet]. c2015 [cited 2015 Dec 21]. Available from: https://www.croix-rouge.fr/Actualite/Lutte-contre-Ebola/Ebola-la-riposte-se-deplace-a-Forecariah-1861

[12] Dixon MG, Schafer IJ Ebola viral disease outbreak – West Africa, 2014. MMWR. 2014;63(25):548–551. 24964881 PMC5779383

[13] World Health Organization Ebola response phase 3: framework for achieving and sustaining a resilient zero [document on the Internet]. c2015 [cited 2015 Dec]. Available from: https://apps.who.int/iris/bitstream/10665/184693/1/ebola_resilientzero_eng.pdf?ua=1

[14] Plucinski MM, Guilavogui T, Sidikiba S, et al Effect of the Ebola-virus-disease epidemic on malaria case management in Guinea, 2014: a cross-sectional survey of health facilities. Lancet Infect Dis. 2015;15(9):1017–1023. 26116183 10.1016/S1473-3099(15)00061-4 PMC4669675

